# A Validated UPLC‐MS/MS Method for Monitoring Temozolomide and Perillyl Alcohol Metabolites in Biological Matrices: Insights From a Preclinical Pharmacokinetic Study

**DOI:** 10.1002/bmc.70253

**Published:** 2025-11-19

**Authors:** Ariane Krause Padilha Lorenzett, Tatiane Patricia Babinski, Samila Horst Peczek, Ana Paula Tartari, Jeferson Ziebarth, Andressa Zago, Thais Carla Brussulo Pereira, Rubiana Mara Mainardes

**Affiliations:** ^1^ Laboratory of Nanostructured Formulations Universidade Estadual do Centro‐Oeste Guarapuava Paraná Brazil; ^2^ Department of Pharmacy Universidade Estadual do Centro‐Oeste Guarapuava Paraná Brazil

**Keywords:** bioanalytical method, bioavailability, biodistribution, glioblastoma

## Abstract

Glioblastoma multiforme (GBM) is among the most aggressive brain tumors, largely due to the restrictive blood–brain barrier (BBB) and limited drug bioavailability. Temozolomide (TMZ), an alkylating agent, and perillyl alcohol (POH), a monoterpene with antitumor properties, have shown promise in GBM therapy. This study developed and validated a UPLC‐MS/MS method for the simultaneous quantification of TMZ, its active metabolite 5‐aminoimidazole‐4‐carboxamide (AIC), and perillic acid (PA), the primary POH metabolite, in rat plasma and tissues. The method met FDA and EMA validation criteria, showing high selectivity, linearity (*R* ^2^ > 0.995), accuracy (80%–120%), precision (RSD < 15%), and matrix‐specific stability across plasma, brain, liver, kidney, spleen, and lung. Following single oral doses of TMZ (2 mg/kg) and POH (47 mg/kg), pharmacokinetic analysis revealed that TMZ had the highest systemic exposure (AUC_0–24h_: 8173.64 ng·h/mL) and C_max_ (1448.64 ng/mL), while PA showed the fastest absorption (t_max_: 0.5 h). AIC levels confirmed efficient TMZ metabolism. Biodistribution analysis showed TMZ accumulation in the brain (~1000 ng/mL), supporting its CNS efficacy. PA was mainly distributed to liver and kidneys, with limited brain penetration. The validated method enables preclinical pharmacokinetic and tissue distribution studies, offering valuable insights into TMZ and POH behavior for GBM treatment.

## 1 Introduction

Malignant brain tumors represent one of the most challenging classes of cancer to treat due to limited accessibility to brain tissues, high mortality rates, and a lack of effective therapeutic options. Among these, glioblastoma is the most aggressive and lethal form, classified as a Grade IV tumor by the World Health Organization (WHO). It is histologically characterized by endothelial proliferation, cerebral necrosis, and an elevated metastatic potential (Louis et al. 2021). Prognosis remains poor, with median survival ranging from 8 to 12 months, depending on tumor location, frontal lobe tumors being associated with comparatively longer survival (Schönthal et al. 2021; Wirsching et al. 2016).

The standard treatment for glioblastoma comprises surgical resection followed by radiotherapy and chemotherapy. Temozolomide (TMZ), an orally administered alkylating agent, is the chemotherapeutic agent of choice. As a prodrug, TMZ exhibits favorable pharmacokinetic properties, including small molecular size and lipophilicity, allowing it to cross the blood–brain barrier (BBB) to some extent. It is rapidly converted into the active metabolites MTIC and AIC, which produce methyldiazonium ions responsible for DNA alkylation, leading to mismatched base pairs, cell cycle arrest at the G2/M checkpoint, and subsequent apoptosis (Lips and Kaina 2001; Tomar et al. 2021). However, the therapeutic efficacy of TMZ is limited by its restricted BBB permeability, with only ~20% of the plasma concentration reaching brain tissues (Ostermann et al. 2004).

In the search for adjunct therapies, perillyl alcohol (POH), a naturally occurring monoterpene found in essential oils from mint, bergamot, and citrus fruits, has gained attention for its potential antitumor activity in brain cancers. Due to its amphiphilic nature, POH can traverse the BBB. Following absorption, it is rapidly metabolized into its major active metabolite, perillic acid (PA). Preclinical studies have demonstrated that POH/PA may induce apoptosis, modulate gene expression, and inhibit oncogenic signaling pathways involved in cell proliferation (Chen et al. 2021; Da Fonseca et al. 2016; Schönthal et al. 2021). Despite these promising findings, the precise mechanisms of POH's antineoplastic action remain poorly elucidated.

Pharmacokinetic and biodistribution studies are essential for elucidating the biopharmaceutical behavior of TMZ and POH; however, they require highly sensitive and selective analytical methods capable of detecting both parent compounds and their metabolites in complex biological matrices. Various analytical approaches, such as HPLC with photodiode array detection (HPLC‐PDA) (European Medicines Agency [EMA] 2021) and UPLC‐MS/MS (Liu et al. 2014; Tiwari and Tiwari 2010; Tudela et al. 2012), have been developed for the individual quantification of TMZ, POH, or their respective metabolites. Furthermore, an HPLC‐PDA method has been reported for the simultaneous analysis of TMZ, POH, and their metabolites derived from the dual‐conjugated compound NEO212 (Andrade et al. 2020). Nonetheless, to date, no UPLC‐MS/MS method, offering superior sensitivity and selectivity over HPLC, has been reported for the simultaneous quantification of TMZ, AIC, and the POH metabolite PA in a single analytical run. Importantly, POH exhibits poor ionization efficiency in mass spectrometry, making its direct detection challenging. However, this limitation is mitigated by the fact that POH is rapidly and extensively converted to PA, which serves as a reliable surrogate marker and pharmacologically active species (Schönthal et al. 2021).

To bridge this analytical gap, the present study aimed to develop, optimize, and validate a UPLC‐MS/MS method for the simultaneous quantification of TMZ, AIC, and PA in rat plasma and tissues. This method was subsequently applied to a pharmacokinetic and biodistribution study following a single oral dose of TMZ and POH in rats. Quantification was performed in plasma and in key organs, including the brain, liver, kidneys, lungs, and spleen, offering critical insight into the systemic and tissue‐specific disposition of these compounds.

## 2 Chemicals and Reagents

TMZ, POH, PA, caffeine, carvone, 5‐aminoimidazole‐4‐carboxamide (AIC), high‐purity formic acid (≥ 98%), and gradient‐grade acetonitrile (ACN, ≥ 99.9%) were purchased from Sigma‐Aldrich (St. Louis, MO, USA). Chromatographic‐grade methanol (LiChrosolv) was obtained from Merck (Darmstadt, Germany).

## 3 Methodology

### 3.1 Preparation of Calibration Standards and Quality Control (QC) Samples

Stock solutions of PA, TMZ, AIC, carvone (internal standard 1, IS1), and caffeine (internal standard 2, IS2) were individually prepared in ACN at a concentration of 1000 μg/mL by accurately weighing 10 mg of each compound into 10 mL volumetric flasks. Subsequently, working solutions at 1000 ng/mL were obtained by diluting 10 μL of the respective stock solution into a separate 10 mL volumetric flask with ACN.

QC samples were prepared by spiking blank rat plasma with PA, AIC, and TMZ at three concentration levels: 30, 80, and 200 ng/mL. Internal standards (IS1 and IS2) were added to each sample at a final concentration of 100 ng/mL. Whole blood was obtained from untreated anesthetized rats that were euthanized as part of the study protocol. Blood samples were collected into heparinized tubes and centrifuged at 3500 rpm for 10 min at 20°C to separate plasma. Organs including the brain, lungs, kidneys, spleen, and liver were promptly excised and homogenized using a Marconi mechanical tissue homogenizer (5 mm probe) in phosphate‐buffered saline (PBS, pH 7.4) at a 1:5 (w/v) tissue‐to‐buffer ratio.

For each matrix sample, 50 μL of homogenate or plasma was mixed with 50 μL of internal standard (IS) solution (100 ng/mL), followed by the addition of the respective working solution to reach the target analyte concentration. The volume was adjusted to 1 mL using an appropriate solvent mixture. Samples were centrifuged again at 3500 rpm for 10 min at 4°C. The resulting supernatants were filtered through 0.22 μm membrane filters prior to chromatographic analysis. All calibration standards and QC samples were prepared freshly on the day of analysis to minimize analyte degradation.

### 3.2 Sample Preparation

Approximately 200 μL of whole blood was collected from the tail vein of each rat. Samples were centrifuged at 3500 rpm for 10 min at 20°C to separate the plasma. Plasma samples were then subjected to liquid–liquid extraction with ACN. The mixture was centrifuged again at 3500 rpm for 10 min at 4°C. The resulting supernatant was filtered through hydrophobic 0.22 μm membranes and transferred to autosampler vials. A 2 μL aliquot of each sample was injected into the UPLC‐MS/MS system for analysis.

Following blood collection, animals were anesthetized and euthanized, and their organs (kidneys, liver, spleen, brain, and lungs) were promptly excised and stored at −20°C. Tissue samples were homogenized in PBS (pH 7.4) at a ratio of 1 part tissue to 5 parts buffer (w/v), using a mechanical homogenizer equipped with a 5 mm probe.

For chromatographic analysis, 200 μL of each tissue homogenate was mixed with 50 μL of IS solution (final IS concentration: 100 ng/mL) and 750 μL of ACN to reach a final volume of 1 mL. The mixture was centrifuged at 3500 rpm for 10 min at 20°C. The supernatant was collected, filtered through a 0.22 μm membrane, and transferred to injection vials for UPLC‐MS/MS analysis. When necessary, samples were diluted appropriately to ensure analyte concentrations fell within the validated linear range.

Thawed plasma samples (50 μL) were also prepared by protein precipitation. Each sample was mixed with 50 μL of IS solution and 900 μL of ACN, followed by centrifugation at 3500 rpm for 10 min at 20°C. The supernatants were filtered and analyzed as described. All dilutions were performed based on analyte concentration to ensure compliance with the method's validated dynamic range.

### 3.3 UPLC‐MS/MS Instrumentation

Chromatographic separation of PA, TMZ, and AIC in rat plasma was achieved using a reverse‐phase C18 column (100 mm × 2.1 mm). The mobile phase consisted of ACN and water containing 0.1% formic acid (60:40, v/v), delivered in isocratic mode at a flow rate of 0.2 mL/min. The total run time for each analysis was 3 min. The injection volume was 2 μL, the column temperature was maintained at 40°C, and the autosampler temperature was set to 10°C to ensure sample stability during analysis.

Detection was performed using a tandem mass spectrometer equipped with an electrospray ionization source operated in positive mode (ESI+). The MS source parameters were as follows: capillary voltage, 4.0 kV; source temperature, 150°C; desolvation temperature, 500°C; and desolvation gas flow, 800 L/h. Quantification was carried out in multiple reaction monitoring (MRM) mode. The optimized mass transitions, cone voltages, and collision energies for each analyte and IS (PA, TMZ, AIC, carvone [IS1], and caffeine [IS2]) are summarized in Table 1.

**TABLE 1:** Mass spectrometric parameters for the analyzed compounds, including mass transitions (m/z), cone voltage (V), and collision energy (eV), for perillic acid (PA), temozolomide (TMZ), 5‐aminoimidazole‐4‐carboxamide (AIC), carvone (IS1), and caffeine (IS2).

Analyte	Retention time (min)	Mass transition (m/z)	Cone voltage (V)	Colision energy (eV)
PA	1.22	167 > 125.01 167 > 92.97	18.0	10
AIC	0.57	126.9 > 110 126.9 > 54.95	18.0	10 20
TMZ	0.75	196.02 > 195.02 195.02 > 137.95	18.0	10
Carvone (IS1)	1.45	151.01 > 122.98 151.01 > 94.99	18.0	10
Caffeine (IS2)	0.69	195.08 > 138.06 195.08 > 110	40	18

**FIGURE 1:**
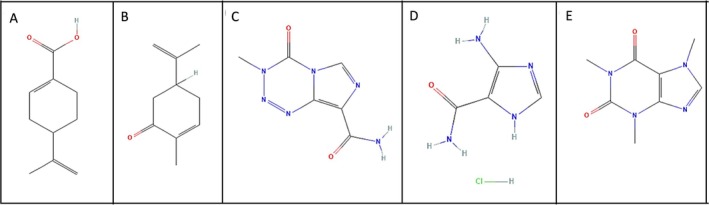
Chemical structures of the analytes and internal standards: (A) perillic acid (PA); (B) carvone (IS1); (C) temozolomide (TMZ); (D) 5‐aminoimidazole‐4‐carboxamide (AIC); and (E) caffeine (IS2).

To ensure accurate quantification, ISs structurally similar to the analytes of interest were selected. Figure 1 illustrates the chemical structures of PA, carvone, TMZ, AIC, and caffeine, supporting their selection based on structural relevance to the target compounds.

### 3.4 Method Validation

The developed method was validated in accordance with the guidelines established by regulatory agencies, including the United States Food and Drug Administration (FDA) and the EMA (2021). The parameters assessed included linearity, selectivity, limit of detection (LOD), limit of quantification (LOQ), precision, accuracy, stability, carryover, and matrix effects to ensure robustness and reliability.

#### 3.4.1 Linearity and Matrix Effect

Calibration curves were constructed over a concentration range of 5–500 ng/mL using seven concentration levels of PA, AIC, and TMZ. Samples were prepared in triplicate in rat plasma, organ homogenates, and ACN, and contained 100 ng/mL of IS1 (carvone) and IS2 (caffeine). The curves were based on the analyte‐to‐IS peak area ratios versus nominal concentrations. Linearity was evaluated by linear regression with a 95% confidence level.

The matrix effect was assessed by comparing the slope of calibration curves prepared in ACN (neat solutions) with those prepared in plasma spiked with the same analyte concentrations (Tudela et al. 2012). The absence of a significant matrix effect was confirmed when no statistically significant differences were observed between the slope coefficients of the curves.

#### 3.4.2 Selectivity

Selectivity was evaluated by analyzing blank matrices to ensure that no endogenous compounds interfered with the detection of the analytes or ISs (Tiwari and Tiwari 2010). Chromatograms of blank samples, spiked samples, and samples containing POH and TMZ were compared to confirm the absence of interfering peaks at the retention times of PA, AIC, TMZ, IS1, and IS2.

#### 3.4.3 LOD and LOQ

LOD was defined as the lowest analyte concentration that could be detected but not necessarily quantified. LOQ was the lowest concentration that could be quantified with acceptable accuracy and precision. These limits were calculated based on the standard deviation (SD) of the y‐intercept (*σ*) and the slope of the calibration curve (*b*), according to Equations (1) and (2):

(1) $$\mathrm{LOD}=\frac{\sigma }{b}x\;3.3$$

(2) $$\mathrm{LOQ}=\frac{\sigma }{b}x\;10$$

where *σ* = SD of the intercept with the y‐axis and *b* = slope of the calibration curve.

#### 3.4.4 Precision and Accuracy

Precision was assessed by repeatability (intra‐day) and intermediate precision (inter‐day), using QC samples at 30, 80, and 200 ng/mL (*n* = 3 per concentration). Intra‐day precision was evaluated on the same day, while inter‐day precision was determined across three consecutive days. Precision was expressed as the relative SD (RSD%), calculated using Equation (3):

(3) $$\mathrm{RSD}=\left(\frac{\mathrm{SD}}{\mathrm{MDC}}\right)x\;100$$

where SD = standard deviation and MDC = mean determined concentration.

Accuracy was expressed as percent recovery, calculated from the ratio between the experimentally determined concentration (EMC) and the theoretical concentration (CT), as shown in Equation (4):

(4) $$\%\text{Recovered}=\frac{\mathrm{EMC}}{\mathrm{CT}}x\;100$$

Triplicate spiked plasma samples were analyzed at the same three concentration levels for accuracy assessment.

#### 3.4.5 Stability

The stability of PA, AIC, and TMZ in rat plasma was assessed under the following conditions using low, medium, and high QC levels: (1) Freeze–thaw stability: Three freeze–thaw cycles over 24‐h intervals; (2) Bench‐top stability: Storage at room temperature for 6 h; (3) Autosampler stability: Storage at 10°C for 24 h. Analytes were considered stable if the relative error (RE%) remained within ±15% (Tiwari and Tiwari 2010).

#### 3.4.6 Carryover Effect

Carryover was evaluated by injecting blank samples immediately after triplicate injections of a high‐concentration standard (250 ng/mL). This assessment was conducted for both plasma and tissue matrices. The response in the blank sample was considered acceptable if it was below 20% of the response observed at the lower LOQ (LLOQ).

### 3.5 Pharmacokinetics and Biodistribution Study

The pharmacokinetic and biodistribution study was conducted in accordance with ethical guidelines and approved by the Animal Ethics Committee of the Universidade Estadual do Centro‐Oeste, Brazil (Protocol No. 026/2023). Male Wistar rats (*n* = 6 for pharmacokinetics; *n* = 4 for biodistribution), weighing approximately 200 g, were housed under controlled environmental conditions (12 h light/dark cycle, 35% relative humidity, and temperature of 23°C ± 2°C). Animals had free access to food and water, which were withheld 10 h prior to drug administration and resumed 2 h afterward.

Each animal received a single oral dose of POH (47 mg/kg) and TMZ (2 mg/kg). For pharmacokinetic profiling, blood samples were collected via the tail vein into heparinized tubes at predefined time points: 0.5, 1, 2, 4, 8, 12, and 24 h post‐administration. For biodistribution analysis, a separate cohort of rats (*n* = 4) was sacrificed 4 h after dosing. Organs, including the brain, liver, lungs, kidneys, and spleen, were collected, homogenized, and stored at −20°C for subsequent quantification, as detailed in Section 3.2.

Pharmacokinetic parameters were calculated using non‐compartmental analysis with the PKSolver plugin for Microsoft Excel. The following parameters were determined: area under the plasma concentration–time curve from 0 to 24 h (AUC_0–24h_), maximum plasma concentration (C_max_), time to reach C_max_ (t_max_), elimination half‐life (t_1/2_), volume of distribution (VD), and total clearance (CL).

### 3.6 Statistical Analysis

All experimental results are expressed as mean ± SD. Statistical comparisons were performed using one‐way analysis of variance (ANOVA), with a confidence level of 95%. A *p*‐value of less than 0.05 was considered statistically significant. Analyses were conducted using GraphPad Prism (GraphPad Software, San Diego, CA, USA).

## 4 Results and Discussion

### 4.1 UPLC‐MS/MS Instrumentation

The analytes evaluated in this study, including TMZ (MW 194.15 g/mol), AIC (MW 126.9 g/mol), PA (MW 166.22 g/mol), as well as the ISs caffeine (MW 194.19 g/mol) and carvone (MW 150.22 g/mol), demonstrated specific and reproducible fragmentation patterns under the optimized MS/MS conditions. The primary mass transitions monitored were: PA: 167 > 92.97, AIC: 126.9 > 110, TMZ: 196.02 > 137.95, caffeine: 195.08 > 138.06, and carvone: 151.01 > 94.99. These fragmentation pathways are consistent with previous reports in the literature, including the work of Liu et al. (2014) for TMZ, (Andrade et al. 2020; Peczek et al. 2023) for PA and carvone, respectively, and Skarkova et al. (2019) for AIC.

ISs were selected based on structural similarity and chromatographic behavior. Caffeine was used as the IS for TMZ and AIC, while carvone was selected for PA. Both IS compounds showed favorable extraction efficiency, retention time compatibility, and ionization profiles, providing robust and consistent detector responses comparable to their respective analytes.

### 4.2 Validation of Bioanalytical Methodology

#### 4.2.1 Linearity and Matrix Effect

Linearity assessment is a fundamental step in analytical method validation, as it confirms that the detector response is directly proportional to the analyte concentration across the working range. This relationship is commonly evaluated through statistical parameters such as the coefficient of determination (*R* ^2^), *p*‐value, and the *F*‐test, which together support the fit of the linear model (Hess and Hess 2017).

As shown in Table 2, linearity was demonstrated for all analytes, TMZ, AIC, and PA across all matrices tested, including plasma, brain, liver, kidneys, spleen, and lungs. In all cases, the *R* ^2^ values exceeded 0.960, indicating excellent correlation between analyte concentration and detector response. Moreover, the *p*‐values for all calibration curves were < 0.05, confirming the statistical significance of the linear regression. Additionally, the calculated *F*‐values were consistently higher than the corresponding critical *F*‐values for each matrix and analyte, further validating the applicability of the linear model and confirming the robustness of the method.

**TABLE 2:** Linearity parameters (*R* ^2^, *p*‐value, *F*‐test) and matrix effect evaluation for the UPLC‐MS/MS method applied to plasma and tissue samples (brain, liver, kidneys, spleen, and lungs).

		Linearity					Matrix effect	
		Regression parameters		Regression				
Matrix	Compound	Equation	*R* ^2^	*p*	*F* calculated	*F* critical	*T* calc.	*T* tab
ACN	PA	*y* = 1.1194*x* + 0.0116	0.990	0.000	3605.84	7.84 × 10^−6^	—	—
	AIC	*y* = 0.266*x* − 1.2328	0.997	0.000	718.38	8.79 × 10^−5^	—	—
	TMZ	*y* = 0.0061*x* + 0.1483	0.992	0.000	86.85	0.0020	—	—
Plasma	PA	*y* = 0.7083*x* + 0.6883	0.993	0.000	8071.78	2.34 × 10^−6^	7.37	4.30
	AIC	*y* = 0.0822*x* − 0.2557	0.996	0.000	1743.09	2.33 × 10^−5^	72.66	4.30
	TMZ	*y* = 0.0034*x* + 0.1387	0.959	0.000	28.38	0.010	56.28	4.30
Brain	PA	*y* = 0.9242*x* − 0.7422	0.995	0.000	30,361.14	3.21 × 10^−7^	3.60	4.30
	AIC	*y* = 0.1360*x* − 0.2059	0.998	0.000	7277.37	2.73 × 10^−6^	36.06	4.30
	TMZ	*y* = 0.0031*x* + 0.1774	0.965	0.000	22.16	0.015	13.73	4.30
Kidney	PA	*y* = 0.7907*x* + 1.0214	0.997	0.000	4729.91	5.22 × 10^−6^	18.47	4.30
	AIC	*y* = 0.091*x* + 0.7661	0.996	0.000	793.82	7.57 × 10^−5^	90.72	4.30
	TMZ	*y* = 0.0033*x* + 0.2495	0.960	0.000	22.10	0.015	39.95	4.30
Lungs	PA	*y* = 0.9084*x* − 3.0936	0.994	0.000	1391.34	0.0007	18.83	4.30
	AIC	*y* = 0.1225*x* + 0.0983	0.999	0.000	14,881.19	9.35 × 10^−7^	91.42	4.30
	TMZ	*y* = 0.0031*x* + 0.1929	0.966	0.000	14.60	0.027	38.35	4.30
Spleen	PA	*y* = 0.8535*x* − 1.1274	0.999	0.000	6717.9	3.08 × 10^−6^	18.58	4.30
	AIC	*y* = 0.1223*x* + 0.7022	0.998	0.000	656.04	0.0001	90.84	4.30
	TMZ	*y* = 0.003*x* + 0.2262	0.979	0.000	14.75	0.027	38.03	4.30
Liver	PA	*y* = 0.8605*x* + 0.3985	0.997	0.000	3836.96	7.14 × 10^−6^	18.32	4.30
	AIC	*y* = 0.1263*x* + 0.2025	0.996	0.000	5683.19	3.96 × 10^−6^	84.75	4.30
	TMZ	*y* = 0.0033*x* + 0.2165	0.966	0.000	14.89	0.026	40.04	4.30

The matrix effect, which refers to signal enhancement or suppression caused by endogenous substances in biological matrices, can significantly influence analytical accuracy and precision. Such interference is particularly relevant in complex matrices like plasma and tissue homogenates (Tudela et al. 2012). In this study, matrix effect evaluation was performed by comparing the slopes of calibration curves in neat solvent (ACN) with those constructed in spiked biological matrices.

The results indicated that for all tested matrices, the calculated *T*‐values exceeded the critical values from the Student's *t*‐distribution table, confirming the presence of a statistically significant matrix effect. These effects are likely attributable to matrix constituents such as proteins, phospholipids, or other endogenous compounds that affect ionization efficiency.

Despite the observed matrix effects, their impact was effectively minimized through the use of structurally appropriate ISs (IS1 and IS2). Furthermore, full validation was conducted separately for each matrix (plasma, brain, kidney, liver, spleen, and lung) to ensure reliable quantification under matrix‐specific conditions.

#### 4.2.2 Selectivity

Selectivity refers to the method's ability to unequivocally identify and quantify the analytes of interest, namely, TMZ, its metabolite AIC, and PA, the primary metabolite of POH, in the presence of endogenous matrix components, such as proteins, metabolites, or other potentially interfering substances. A selective method must ensure that the analyte peaks are well resolved from the blank matrix and ISs, thereby preventing analytical bias and maintaining precision and accuracy (EMA 2021).

**FIGURE 2:**
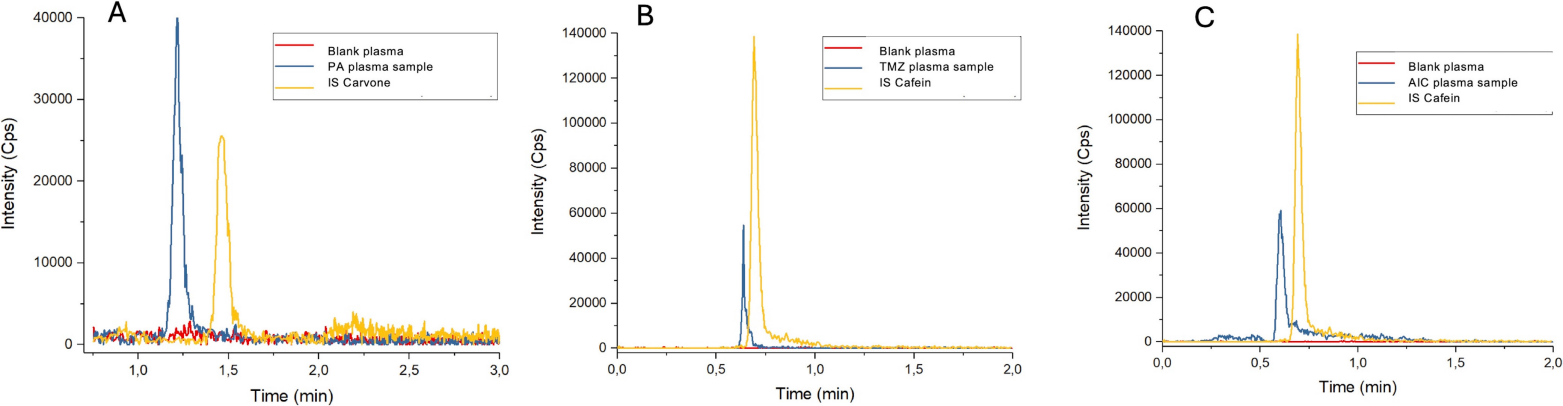
Representative chromatograms of (A) perillic acid (PA), (B) temozolomide (TMZ), and (C) 5‐aminoimidazole‐4‐carboxamide (AIC) in rat plasma.

**FIGURE 3:**
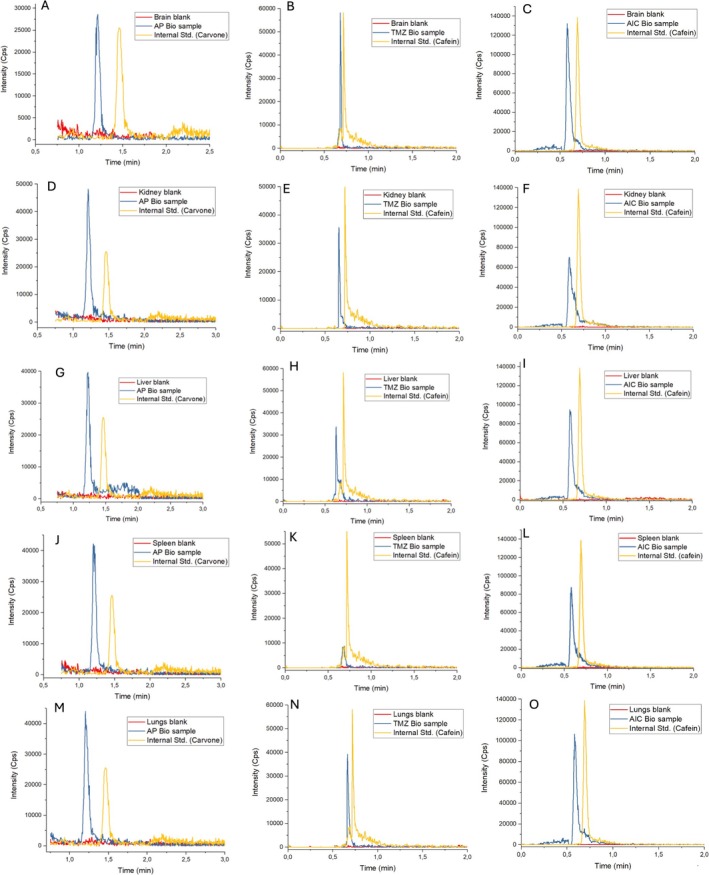
Representative chromatograms of perillic acid (PA), temozolomide (TMZ), and 5‐aminoimidazole‐4‐carboxamide (AIC) in various rat tissues: brain (A–C), kidney (D–F), liver (G–I), spleen (J–L), and lung (M–O).

**FIGURE 4:**
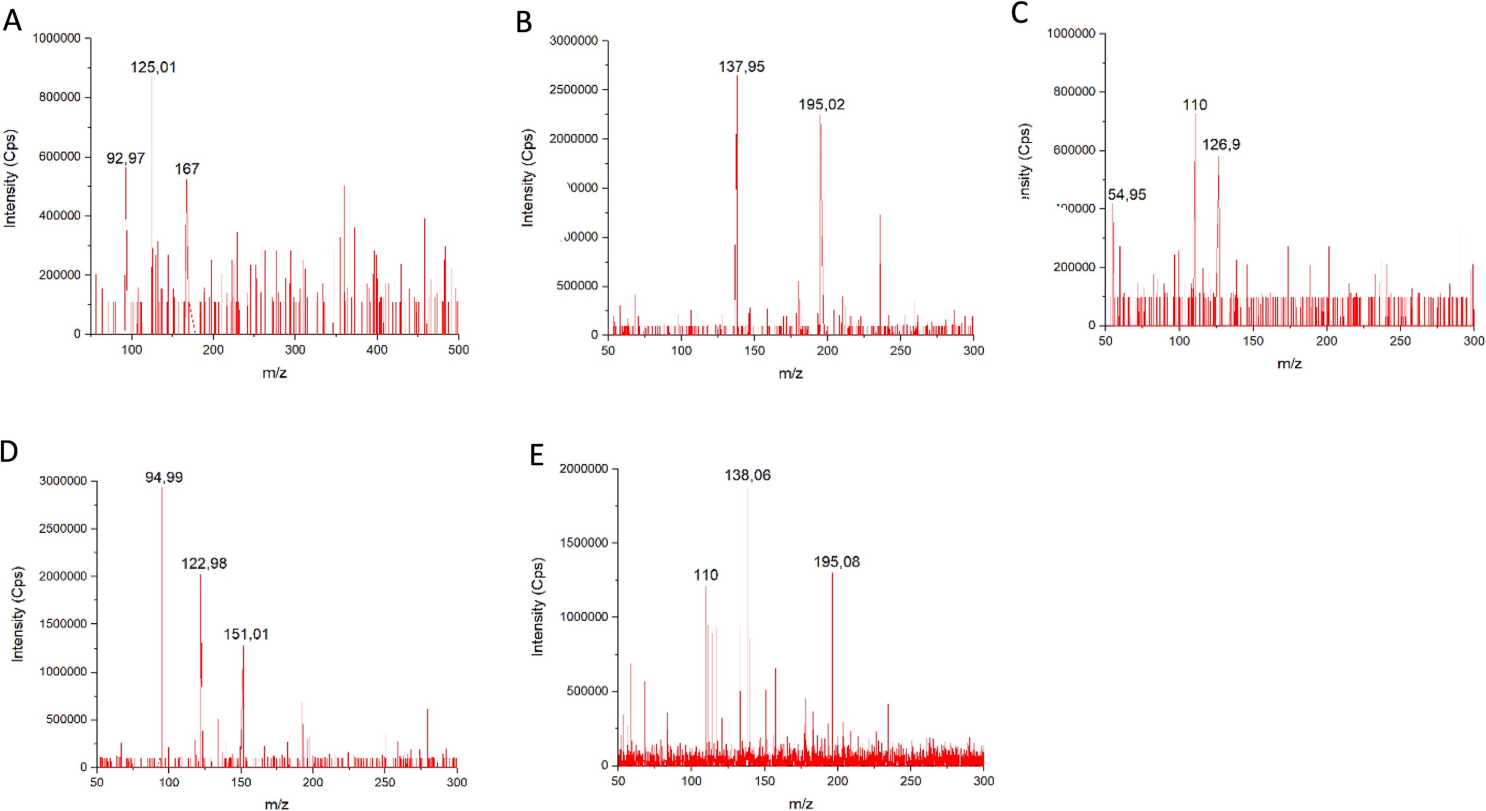
Mass spectra of (A) perillic acid (PA), (B) temozolomide (TMZ), (C) 5‐aminoimidazole‐4‐carboxamide (AIC), (D) carvone (IS1), and (E) caffeine (IS2) in acetonitrile, showing the major fragmentation ions used for quantification in MRM mode.

Figures 2 and 3 illustrate representative chromatograms demonstrating selectivity in plasma (Figure 2A–C) and various tissues, including spleen, kidney, liver, lung, and brain (Figure 3A–O). Additionally, Figure 4 shows the mass spectra of each analyte, with clear identification of their respective fragmentation ions.

Across all tested matrices, no interfering peaks were observed at the retention times of the target analytes or ISs. The chromatographic profiles exhibited adequate resolution and signal intensity, indicating that the developed UPLC‐MS/MS method meets the selectivity requirements outlined by regulatory guidelines.

The use of ISs, caffeine for TMZ and AIC, and carvone for PA, further ensured consistency in recovery and ionization efficiency across different matrices. The application of matrix‐specific controls and the separate validation of each tissue type underscore the method's robustness and adaptability to the complexity and variability of biological samples.

#### 4.2.3 Carryover

Carryover refers to the residual presence of analytes from a previous sample that may interfere with the analysis of subsequent injections. This phenomenon is particularly relevant in automated analytical systems or when inadequate cleaning of the injection system occurs between samples. Carryover can lead to falsely elevated concentrations, especially when analyzing samples with low analyte levels following high‐concentration injections (Kristensen et al. 2022).

In accordance with bioanalytical validation guidelines, acceptable carryover levels must not exceed 20% of the response observed at the LLOQ. In the present study, carryover was evaluated in ACN, plasma, and various tissue matrices. No carryover was detected for any of the analytes, PA, AIC, or TMZ, in ACN.

In plasma, the carryover levels were within acceptable limits: 9.90% for PA, 15.87% for AIC, and 5.47% for TMZ. For brain tissue, values were 7.21% (PA), 0.85% (AIC), and 3.37% (TMZ). In kidney homogenates, carryover was 1.67% for PA, 6.26% for AIC, and 2.72% for TMZ. In lung tissue, PA showed the highest carryover at 14.47%, followed by AIC at 8.10% and TMZ at 4.82%. Lastly, in spleen samples, carryover was 1.50% (PA), 5.88% (AIC), and 5.86% (TMZ).

#### 4.2.4 LOQ and LOD

The LOQ and LOD values obtained for each analyte in the different biological matrices are summarized in Table 3. These parameters reflect the sensitivity of the analytical method and its ability to detect and quantify low analyte concentrations.

**TABLE 3:** Limit of detection (LOD) and limit of quantification (LOQ) of PA, AIC, and TMZ in plasma and tissue matrices using the validated UPLC‐MS/MS method.

LOQ (ng/mL)				LOD (ng/mL)		
PA		AIC	TMZ	PA	AIC	TMZ
ACN	34.87	2.55	0.53	11.51	0.84	0.18
Plasma	15.02	2.00	1.26	4.96	0.66	0.42
Brain	16.75	12.40	5.54	5.53	4.09	1.83
Spleen	12.45	3.94	14.34	4.11	1.30	4.73
Liver	20.75	17.33	21.79	6.85	5.72	7.19
Kidney	7.98	23.78	18.18	2.63	7.84	6.00
Lungs	20.47	7.34	26.24	6.75	2.42	8.66

In plasma, the method demonstrated high sensitivity for AIC (LOQ of 2.00 ng/mL and LOD of 0.66 ng/mL) and TMZ (LOQ of 1.26 ng/mL and LOD of 0.42 ng/mL). In contrast, the LOQ for PA was higher (15.2 ng/mL), indicating that the method requires relatively higher concentrations for accurate quantification of this compound. These differences in sensitivity are consistent with the physicochemical properties and ionization efficiencies of the analytes.

The low LOD values for AIC across all matrices confirm the method's capacity to detect trace concentrations of this metabolite, which is particularly relevant for pharmacokinetic and tissue distribution studies where analyte levels may be low.

In tissue matrices such as spleen and liver, both LOQ and LOD values for all analytes were generally higher compared with other tissues. For instance, TMZ in the liver exhibited an LOQ of 21.79 ng/mL and an LOD of 7.19 ng/mL. These elevated values likely reflect the higher complexity and greater interference potential of these tissues, which may hinder ionization efficiency and impact analytical sensitivity.

Conversely, LLOQ and LOD values were observed in brain and plasma samples, suggesting enhanced sensitivity of the method in these matrices. For example, PA in the kidney showed an LOQ of 7.98 ng/mL and an LOD of 2.63 ng/mL, demonstrating the method's capacity to detect and quantify low analyte levels in these tissues with high reliability.

Overall, despite some variability across matrices, the results confirm that the developed UPLC‐MS/MS method is sensitive and well‐suited for quantifying low concentrations of PA, AIC, and TMZ in both plasma and tissue samples, supporting its application in pharmacokinetic and biodistribution studies.

#### 4.2.5 Precision, Accuracy, and Stability

As summarized in Table 4, the method demonstrated satisfactory intra‐ and inter‐assay precision across all tested matrices. The RSD values were below the generally accepted limit of 15%, as recommended by regulatory agencies. For example, plasma samples showed RSDs ranging from 0.74% to 12.94%. Although slightly higher variability was observed in brain tissue, particularly for TMZ at the 30 ng/mL level (10.01%), all results remained within the acceptable threshold. These findings confirm that the method consistently yields reproducible results, even in more complex matrices.

**TABLE 4:** Precision (%RSD), accuracy (% recovery), and stability results for PA, AIC, and TMZ in plasma and tissue matrices. Stability was evaluated under freeze–thaw, bench‐top, and autosampler conditions.

Matrix	Compound	Concentration (ng/mL)	Precision (RSD %)		Accuracy (%)		Stability (ER %)		
			Intra‐day	Inter‐day	Recovery	RSD	Freeze/thaw	Bench‐top	Autosampler
Plasma	PA	30	2.95	9.37	103.71	12.43	1.26	5.32	10.61
		80	0.74	5.83	109.32	3.46	8.67	11.90	14.01
		200	0.96	3.09	110.99	12.48	4.45	13.82	3.21
	AIC	30	6.23	7.93	112.35	3.42	10.24	6.77	12.36
		80	5.32	5.30	101.93	6.39	5.34	10.93	13.46
		200	4.57	8.53	103.04	5.12	1.04	9.03	3.48
	TMZ	30	12.94	11.29	97.46	13.42	3.94	1.39	12.84
		80	4.30	6.94	99.03	1.45	4.29	3.94	5.39
		200	4.93	5.23	102.92	2.58	9.83	1.94	13.24
Brain	PA	30	8.46	5.17	102.08	11.33	1.93	1.28	2.95
		80	3.98	9.56	101.26	12.84	7.85	10.03	2.37
		200	9.85	7.45	104.73	1.13	2.46	5.74	4.49
	AIC	30	6.60	9.67	97.88	13.21	8.55	7.13	2.04
		80	8.57	6.46	104.51	10.73	1.79	8.22	4.29
		200	2.59	7.78	97.75	1.05	6.76	9.26	4.57
	TMZ	30	10.01	5.14	105.24	11.71	9.76	1.48	1.65
		80	9.86	5.32	101.45	8.74	0.84	7.73	1.89
		200	4.90	6.74	107.34	1.36	4.42	2.32	4.29
Liver	PA	30	1.98	5.50	105.69	14.62	1.52	1.59	5.04
		80	0.55	5.75	108.81	13.88	3.24	2.49	2.92
		200	7.80	5.65	103.02	9.22	2.39	0.42	4.45
	AIC	30	3.60	6.55	106.85	13.05	1.67	9.13	4.35
		80	8.97	4.75	101.56	10.75	9.53	3.28	1.74
		200	2.64	9.18	99.62	11.05	7.67	8.71	2.34
	TMZ	30	8.74	6.82	103.23	12.24	5.67	1.49	3.82
		80	1.38	7.21	97.66	11.62	8.17	6.41	4.07
		200	9.16	3.99	105.11	9.98	1.54	1.57	3.33
Kidney	PA	30	5.50	10.16	101.01	10.29	8.61	9.61	4.24
		80	1.07	6.83	101.09	12.86	7.99	7.75	3.42
		200	1.12	5.23	104.94	11.71	1.82	1.36	3.31
	AIC	30	0.58	4.73	107.83	12.81	1.29	2.11	4.28
		80	1.07	9.31	98.45	10.56	8.45	8.94	5.42
		200	1.33	8.63	99.54	11.81	6.46	8.76	5.94
	TMZ	30	11.34	6.13	104.53	10.32	3.69	1.99	1.41
		80	2.19	8.75	100.57	9.43	9.44	8.22	3.14
		200	9.64	4.38	108.89	1.45	4.52	3.77	4.92
Lungs	PA	30	8.99	4.37	102.42	10.25	2.68	1.39	4.01
		80	10.05	4.03	103.81	12.27	4.23	2.94	4.14
		200	3.49	7.01	100.34	11.47	6.65	8.02	3.04
	AIC	30	4.38	6.48	100.01	10.04	5.74	7.93	2.22
		80	9.12	5.11	101.73	10.62	1.04	8.37	2.84
		200	3.46	9.97	98.53	9.42	6.67	8.49	3.33
	TMZ	30	1.19	10.32	99.33	12.63	6.24	7.99	4.82
		80	3.98	9.32	97.66	10.86	7.19	9.32	3.02
		200	3.33	7.66	107.96	14.43	1.34	5.54	2.51
Spleen	PA	30	0.64	5.05	108.92	1.50	2.72	2.55	3.25
		80	1.78	7.45	98.92	12.29	5.52	6.84	2.24
		200	1.35	10.38	98.21	9.33	8.51	9.57	3.41
	AIC	30	1.22	5.91	107.64	1.50	8.36	1.42	4.01
		80	1.48	6.48	107.92	1.93	4.43	3.74	4.98
		200	10.97	6.32	103.64	8.82	1.32	1.52	3.13
	TMZ	30	3.94	10.08	98.28	9.54	5.86	6.41	2.64
		80	9.12	6.23	102.89	11.04	1.35	1.35	2.61
		200	2.27	6.84	108.69	1.55	3.86	1.05	2.22

Accuracy, assessed through recovery studies, also met validation criteria, with values ranging between 80% and 120% and RSDs below 15%. The lowest recovery was observed for AIC at 200 ng/mL in brain tissue (97.75%), while the highest occurred for AIC at 30 ng/mL in the plasma (112.34%). These data indicate that the method can accurately quantify the analytes without significant matrix‐related losses or artifacts.

Stability assessments were conducted to ensure that the analytes remained chemically and physically stable under routine sample handling and storage conditions. According to the criteria, a variation of up to ±15% relative to the initial concentration is considered acceptable. The results, shown in Table 4, demonstrated excellent analyte stability, with ER values ranging from 0.84% to 14.01% across all matrices and conditions tested. These findings confirm the robustness of the method for pharmacokinetic and biodistribution studies, supporting reliable quantification even after extended handling or sample processing.

### 4.3 Pharmacokinetic Parameters and Biodistribution Study

**FIGURE 5:**
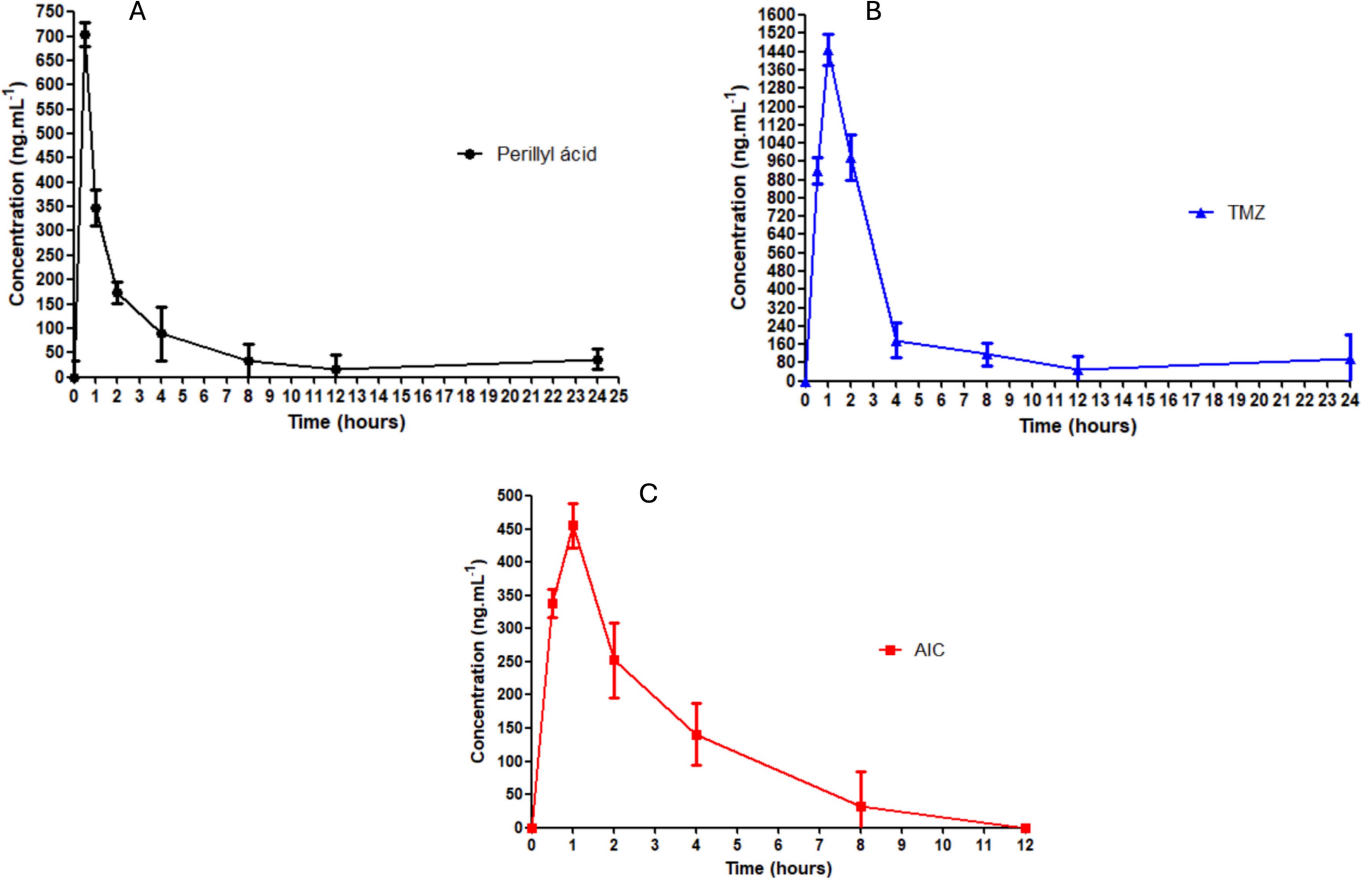
Mean plasma concentration–time curves (±SD) of (A) perillic acid (PA), (B) temozolomide (TMZ), and 5‐aminoimidazole‐4‐carboxamide (C) AIC following single oral administration of POH and TMZ in rats (*n* = 6).

The validated UPLC‐MS/MS method was applied to characterize the pharmacokinetics and tissue distribution of TMZ, AIC, and PA following single oral administration in rats. The mean plasma concentration–time profiles are shown in Figure 5, and corresponding pharmacokinetic parameters are presented in Table 5.

**TABLE 5:** Pharmacokinetic parameters of PA, AIC, and TMZ in rats following single oral administration of POH and TMZ (*n* = 6).

Parameter	PA	AIC	TMZ
C_max_ (ng/mL)	704.2	2187.54	1448.64
t_max_ (h)	0.5	1.0	1.0
AUC_0–24h_ (ng·h/mL)	2198.12	2187.54	8173.64
t_1/2_ (h)	19.04	18.48	18.44
Kel (1/h)	0.036	0.037	0.037
Vd (L/kg)	6.26	6.09	1.75
CL (L/h/kg)	0.23	0.23	0.09

The differences in absorption and exposure are consistent with previous findings. TMZ's rapid degradation under physiological pH and efficient conversion to AIC under acidic tumor‐like conditions explain its targeted efficacy in glioblastoma (Petrenko et al. 2022; Pourmasoumi et al. 2024; Zhang et al. 2012). Conversely, PA's rapid absorption but lower AUC is characteristic of lipophilic molecules with high plasma protein binding and fast clearance (Chen et al. 2021; Cho et al. 2012; Schönthal et al. 2022).

Additionally, TMZ's low VD supports its accumulation in the central compartment, favoring CNS penetration, a key attribute for glioblastoma therapy. These findings are consistent with prior pharmacokinetic studies of TMZ analogs, which associate limited tissue distribution with effective brain targeting (Meadows et al. 2002; Minea et al. 2024; Peczek et al. 2023).

**FIGURE 6:**
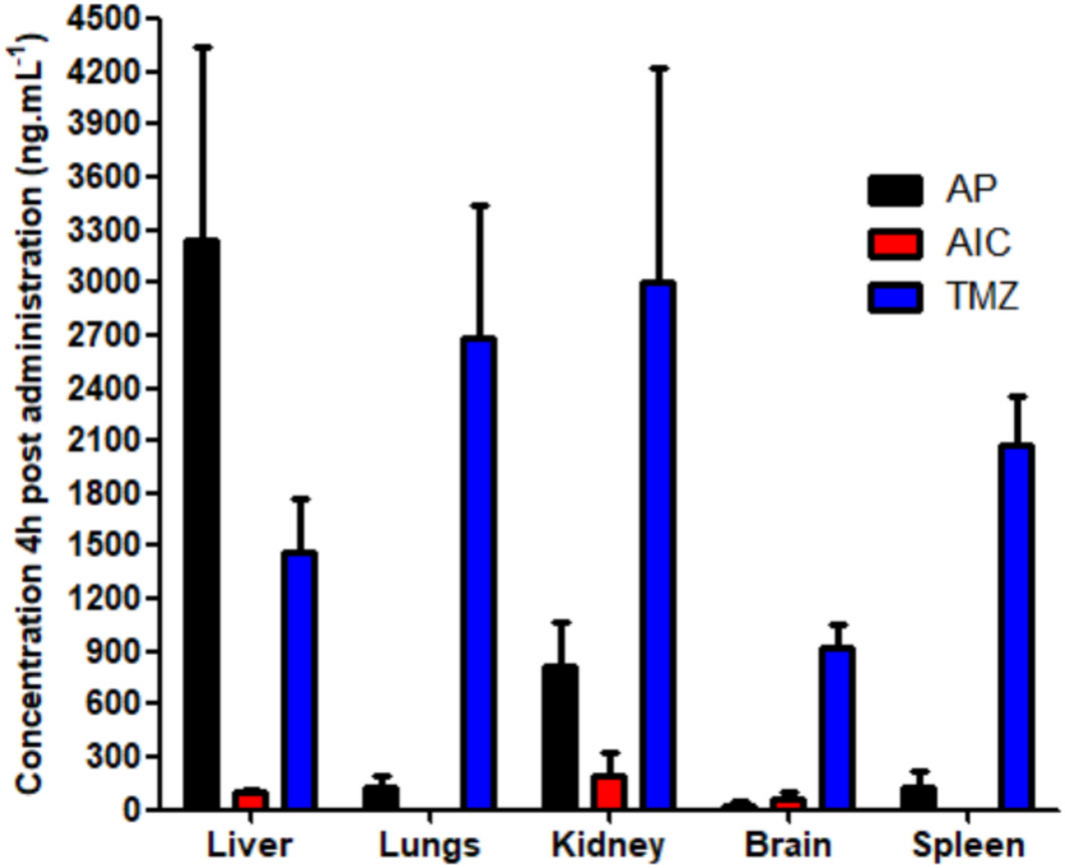
Mean tissue concentrations of perillic acid (PA), 5‐aminoimidazole‐4‐carboxamide (AIC), and temozolomide (TMZ) in brain, liver, spleen, kidney, and lung 4 h after single oral administration of POH and TMZ (*n* = 4).

Biodistribution data obtained 4 h after administration (Figure 6) confirmed differential tissue penetration across the analytes. TMZ showed the highest tissue concentrations in all organs, especially the liver, kidneys, and spleen, consistent with its larger AUC and restricted distribution volume. This suggests preferential localization in metabolically active and excretory tissues.

PA demonstrated marked hepatic accumulation, likely due to extensive first‐pass metabolism (Wang et al. 2021). However, its levels in the brain, lungs, and kidneys were significantly lower compared with TMZ, likely reflecting rapid elimination. The early t_max_ of PA (within 30 min) may also contribute to its limited tissue retention.

AIC, as a downstream TMZ metabolite, showed the lowest tissue concentrations, reinforcing its dependence on TMZ bioactivation. Both C_max_ and AUC values for AIC were significantly lower than those for TMZ, as expected from its secondary metabolic origin.

Importantly, TMZ exhibited the highest concentration in the brain, confirming its capacity to cross the BBB and supporting its clinical utility in CNS‐targeted therapies such as glioblastoma (Friedman et al. 2000). This contrasts with PA and AIC, whose lower brain concentrations suggest more limited CNS penetration.

Furthermore, the relatively high concentrations of TMZ and PA in the kidneys indicate active renal elimination pathways, while the prominent hepatic levels, particularly for PA highlight the role of liver metabolism in their clearance (Bruinsmann et al. 2019; Reyderman et al. 2004).

Taken together, the pharmacokinetic and biodistribution data highlight distinct profiles for TMZ, AIC, and PA in terms of absorption, tissue penetration, and elimination. The higher brain concentrations achieved by TMZ, along with its favorable systemic exposure, reinforce its relevance in CNS‐targeted therapies. In contrast, PA's rapid absorption but limited CNS distribution suggests potential utility in peripheral tumors or in combination regimens. These findings support the continued investigation of these agents and underscore the value of the validated analytical method for future preclinical evaluations.

## 5 Conclusion

A sensitive and validated UPLC‐MS/MS method was developed for the simultaneous quantification of TMZ, its metabolite AIC, and PA in rat plasma and tissues. The method met all regulatory validation criteria and was successfully applied to a pharmacokinetic and biodistribution study in rats. Results revealed distinct pharmacokinetic profiles, with TMZ showing the highest systemic exposure and brain penetration, supporting its therapeutic use in glioblastoma. PA demonstrated rapid absorption but faster clearance, while AIC reflected TMZ biotransformation. These findings contribute valuable insight into the biopharmaceutical behavior of TMZ and POH and provide a reliable analytical tool for future preclinical investigations. The validated method offers broad applicability for pharmacokinetic, biodistribution, and potentially drug interaction studies involving TMZ, POH, and related analogs in complex biological systems.

## Author Contributions

**Ariane Krause Padilha Lorenzett:** conceptualization, writing – review and editing, methodology, practical experiments, visualization and supervision. **Tatiane Patricia Babinski:** review and editing, practical experiments. **Jeferson Ziebarth:** review and editing, practical experiments. **Samila Horst Peczek:** review and editing, practical experiments. **Ana Paula Tartari:** review and editing, practical experiments. **Andressa Zago:** practical experiments. **Thais Carla Brussulo Pereira:** practical experiments. **Rubiana Mara Mainardes:** term, formal analysis, writing – review and editing, supervision and project administration.

## Conflicts of Interest

The authors declare no conflicts of interest.

## Data Availability

The datasets generated and/or analyzed during the current study are available from the corresponding author on reasonable request.
